# P017: Reduction of catheter associated urinary tract infections following removal of unnecessary urinary catheters in a tertiary care hospital in Saudi Arabia

**DOI:** 10.1186/2047-2994-2-S1-P17

**Published:** 2013-06-20

**Authors:** DA Abdulmutalib, AT Abato, W Mazi, A Senok

**Affiliations:** 1 infection prevention and control, King Abdul Aziz Specialist Hospital, Taif, Saudi Arabia; 2 College of Medicine, Alfaisal University, Riyadh, Saudi Arabia

## Introduction

Reduction of catheter associated urinary tract infections (CAUTI) is important in improving rates of hospital associated infections as apatient safety.

## Objectives

This study aimed to assess the impact of the Society for Healthcare Epidemiology of America/ Infectious Diseases Society of America (SHEA/IDSA) practice recommendations for removal of unnecessary urinary catheters to prevent CAUTI.

## Methods

The prospective study was conducted from January 2011-March 2013 at the Medical/Surgical wards in King Abdul Aziz Specialist Hospital, Taif, Saudi Arabia. CAUTI was identified using the Centers for Disease Control and Prevention criteria. Baseline CAUTI rates was collected in 2011 and in 2012 a CAUTI-Team was established to remove unnecessary urinary catheters following SHEA/IDSA practice recommendations. To enable benchmarking with National Healthcare Safety Network (NHSN, USA), data collection and analysis were carried out in accordance with NHSN recommendations (DA-Module 2010).

## Results

The incidence of CAUTI declined from 3.5 to 2.9 per 1000 catheter-days in 2011 and 2012 with further reduction to 2.2 per 1000 catheter-days in the first quarter of 2013. Benchmarking with NHSN, the incidence rate of CAUTI was above 75^th^-90^th^ percentile, while the utilization ratio was 50^th^ to 75^th^ percentile of NHSN hospitals in 2011.After implementation of removing unnecessary urinary catheters, the incidence rate declined 15% in 2012 (Standardized Infection Ratio (SIR) 0.85). Compared to 2011, the reduction rates observed during the fourth quarter of 2012 and first quarter of 2013 (1.7 and 2.5/1000 catheter-days, respectively). *Escherichia coli* and *Pseudomonas aeruginosa* were the most common organisms causing CAUTI.

## Conclusion

Our findings indicate the beneficial role of removal unnecessary urinary catheters for reduction of CAUTI rates in our setting.

## Disclosure of interest

None declared

